# ATP-dependent substrate transport by the ABC transporter MsbA is proton-coupled

**DOI:** 10.1038/ncomms12387

**Published:** 2016-08-08

**Authors:** Himansha Singh, Saroj Velamakanni, Michael J. Deery, Julie Howard, Shen L. Wei, Hendrik W. van Veen

**Affiliations:** 1 Department of Pharmacology, University of Cambridge, Tennis Court Road, Cambridge CB2 1PD, UK; 2 Department of Biochemistry, Cambridge Centre for Proteomics, University of Cambridge, Tennis Court Road, Cambridge CB2 1GA, UK

## Abstract

ATP-binding cassette transporters mediate the transbilayer movement of a vast number of substrates in or out of cells in organisms ranging from bacteria to humans. Current alternating access models for ABC exporters including the multidrug and Lipid A transporter MsbA from *Escherichia coli* suggest a role for nucleotide as the fundamental source of free energy. These models involve cycling between conformations with inward- and outward-facing substrate-binding sites in response to engagement and hydrolysis of ATP at the nucleotide-binding domains. Here we report that MsbA also utilizes another major energy currency in the cell by coupling substrate transport to a transmembrane electrochemical proton gradient. The dependence of ATP-dependent transport on proton coupling, and the stimulation of MsbA-ATPase by the chemical proton gradient highlight the functional integration of both forms of metabolic energy. These findings introduce ion coupling as a new parameter in the mechanism of this homodimeric ABC transporter.

## Abstract (web summary)

ABC exporters mediate the translocation of cytotoxic compounds to the cell exterior via ATP hydrolysis. Here, the authors show that the bacterial transporter MsbA requires additional energy from the transmembrane electrochemical proton gradient to facilitate drug transport.

## Graphical abstract

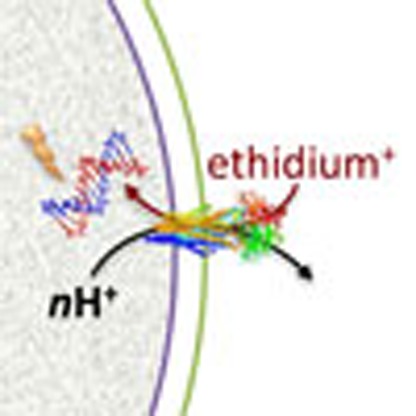

ATP-binding cassette (ABC) multidrug exporters are embedded in the plasma membrane and actively extrude cytotoxic drugs from the cell^1^. They play a critical role in the failure of pharmacological treatment of microbial diseases and cancers, affect drug pharmacokinetics in mammals and are a prime target for clinical research^2^,^3^. Some of these transporters, including the mammalian multidrug resistance P-glycoprotein ABCB1 and its bacterial homologues MsbA and LmrA, transport lipids and chemotherapeutic drugs from the inner leaflet of the plasma membrane to the outer leaflet and extracellular environment^4^,^5^,^6^,^7^,^8^.

ABC exporters are thought to utilize the free energy from ATP-binding and hydrolysis at two nucleotide-binding domains (NBDs) to transport substrates via a translocation pathway that is formed by two membrane domains (MDs)^9^,^10^. In ABCB1, these four domains are fused on a single polypeptide, whereas in bacterial MsbA and LmrA, an MD is fused to an NBD in a half-transporter that homodimerizes to form the full transporter. Current structural and biochemical data support an ‘alternating access' model in which the substrate-binding sites in the MDs are exposed to either side of the membrane as the transporter alternates between inward-facing and outward-facing conformational states^11^,^12^,^13^. The transition from the inward-facing conformation to the outward-facing conformation is governed by ATP-binding-associated NBD dimerization, often referred to as ‘the power stroke', after which ATP hydrolysis and ADP-and-Pi-release-dependent NBD dissociation resets the transporter to the inward-facing conformation. However, many important details of this mechanism remain to be elucidated. MsbA transports cytotoxic agents and the Lipid A anchor of lipopolysaccharides^14^,^15^,^16^,^17^, and is an essential transporter in many Gram-negative bacteria^18^,^19^,^20^. Here we show for *Escherichia coli* MsbA that ATP binding and hydrolysis are insufficient to drive drug transport in the absence of an electrochemical proton gradient. We conclude that proton coupling is essential in the nucleotide-dependent power stroke in MsbA.

## Results

### Studies in intact cells

**Figure 1:**
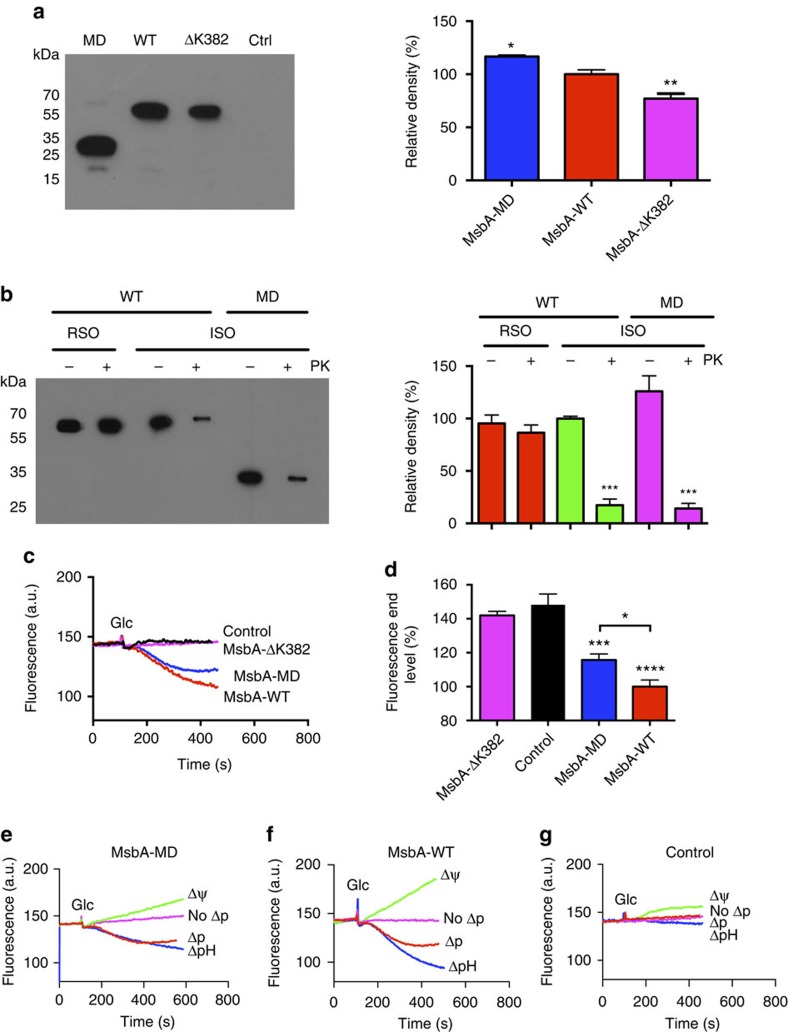
Ethidium efflux in intact cells. (**a**) Immunoblot probed with anti-polyhistidine tag antibody (left) shows that MsbA-MD and MsbA-ΔK382 are expressed in the plasma membrane of *L. lactis* (5 μg total membrane protein per lane) at 117% and 77% of MsbA-WT, respectively, and that these proteins are absent in control cells (Ctrl). The migration of molecular mass markers is indicated. Histogram (right) shows MsbA signal intensities. (**b**) Availability of the cytosolic NH_2_-terminal His-tag in MsbA-WT and MsbA-MD to cleavage by proteinase K (+PK) at the external side of right-side-out (RSO) or inside-out (ISO) membrane vesicles (3 μg protein per lane). Incubation without the protease (-PK) served as control. Uncleaved His-tag was detected on immunoblot (left). Signal intensities are shown in the histogram (right). (**c**) Efflux of monovalent cationic ethidium was initiated by the addition of 20 mM glucose (Glc) as a source of metabolic energy to ATP-depleted cells that were preloaded with 2 μM of the dye. Efflux was observed for MsbA-WT but not for non-expressing control or MsbA-ΔK382, which exhibits a strongly reduced ATPase activity due to the absence of the catalytic Walker A lysine residue. Remarkably, ethidium efflux was also observed for a truncated form of MsbA-WT that lacks the NBD (MsbA-MD). (**d**) Histogram shows significance of fluorescence levels in (**c**) at *t*=400 s. (**e–g**) Ethidium efflux from cells containing MsbA-MD (**e**), MsbA-WT (**f**) or no MsbA proteins (g) to which ionophores nigericin (Δ*ψ* only, interior negative), valinomycin (ΔpH only, interior alkaline) or both (no Δ*p*) were added at concentrations of 1.0 and 0.1 μM, respectively, 3 min prior to the addition of the glucose. Data represent observations in 3 or more independent experiments with independently prepared batches of cells. Values in histograms are expressed as mean±s.e.m. (one-way analysis of variance; *P<0.05; **P<0.01; ***P<0.001; ****P<0.0001).

Energy coupling by MsbA was first studied in ATP-depleted *Lactococcus lactis* cells with a very low internal ATP concentration of ∼7 μM (ref. 21) that were preloaded with 2 μM ethidium by reversed transport by MsbA^15^,^16^ (Fig. 1). After a steady state was reached, the addition of glucose raised the intracellular ATP concentration to ∼9 mM (ref. 21), and initiated a significant ethidium efflux activity by wild-type MsbA (MsbA-WT) compared with the non-expressing control (Fig. 1c,d). Surprisingly, ethidium efflux was also observed for cells containing MsbA-MD (Fig. 1c,d), a truncated form of MsbA-WT that lacks the NBD and that is expressed in a similar orientation and at a moderately elevated level (117%) in the plasma membrane compared with MsbA-WT (Fig. 1a,b). To investigate the possibility that transport by MsbA-MD in these cells is dependent on an electrochemical proton gradient, also referred to as the protonmotive force (Δ*p*, interior positive and acidic), or one of its components, the transmembrane pH gradient (ΔpH) and electrical membrane potential difference (Δ*ψ*), measurements of ethidium efflux by MsbA-MD were repeated in cells in which the magnitude and composition of the Δ*p* (=Δ*ψ*−*Z*ΔpH in which *Z* is approximately equal to 58 mV at 20 °C) was manipulated with the ionophores nigericin and valinomycin^22^. The results show that ethidium efflux by MsbA-MD was completely inhibited in the presence of the Δ*ψ* only. In contrast, significant efflux was observed in the presence of the ΔpH only (Fig. 1e). The results for MsbA-WT (Fig. 1f) showed similarities with those for MsbA-MD, and both were clearly different from non-expressing control cells for which no ethidium efflux was observed (Fig. 1g). Previous studies in cells highlighted the dependency of ethidium efflux by MsbA-WT on ATP binding and hydrolysis; the efflux activity is strongly inhibited by impairment of the MsbA-ATPase activity down to 4–6% of WT activity through the deletion of the Walker A lysine residue at position 382 (ΔK382 mutation)^16^,^23^. Indeed, although the expression level of MsbA-ΔK382 was only slightly below that of MsbA-WT (77%; Fig. 1a), the ethidium transport activity of the mutant was strongly inhibited (Fig. 1c,d). Taken together, these findings suggest that MsbA-mediated ethidium efflux is dependent on both the electrochemical proton gradient and ATP hydrolysis.

### Proton-coupled substrate transport in proteoliposomes

**Figure 2:**
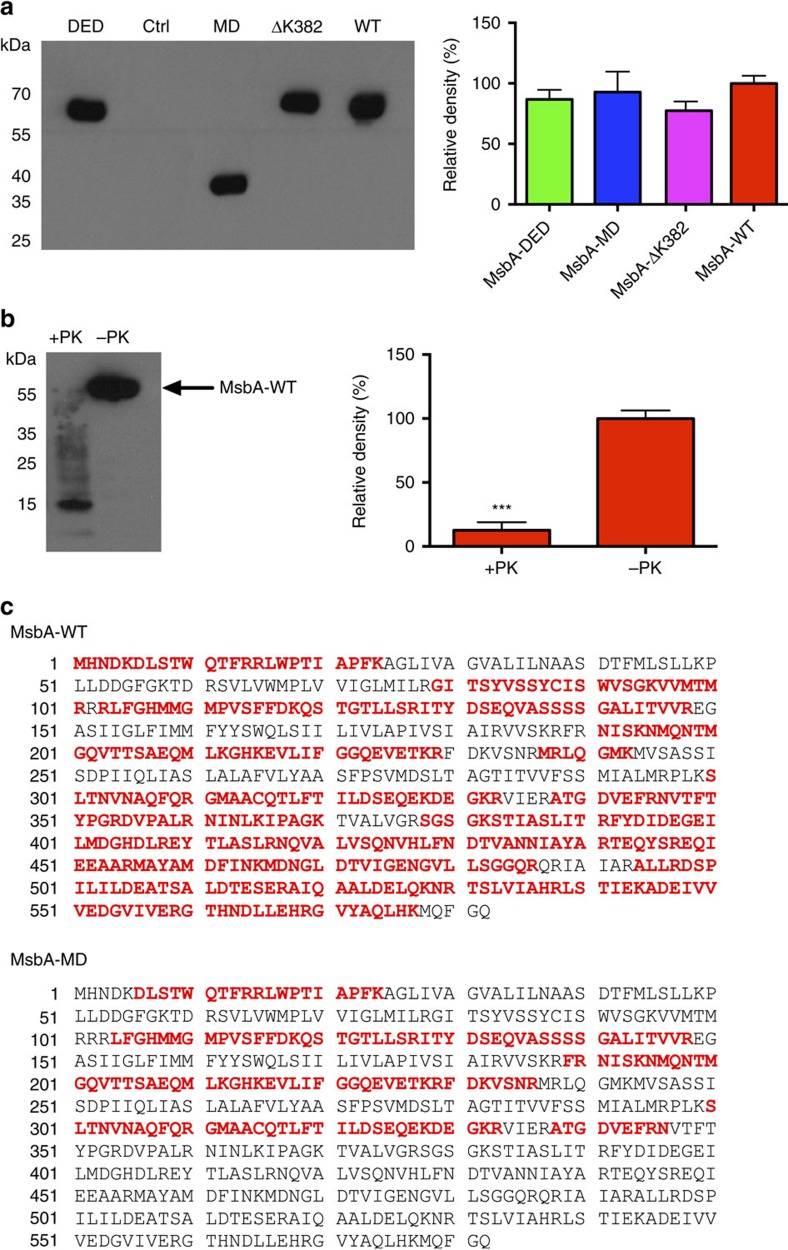
Purified MsbA proteins in proteoliposomes. (**a**) Immunoblot of proteoliposomes prepared from *E. coli* phospholipids (5 μg protein per lane) (left) demonstrates the equal incorporation of purified transport-inactive triple mutant MsbA-DED, MsbA-MD, MsbA-ΔK382 and MsbA-WT, and the absence of membrane proteins in empty control liposomes (Ctrl). Histogram (right) shows MsbA signal intensities. (**b**) Availability of the His-tag in MsbA-WT (3 μg protein per lane) to cleavage by Proteinase K (PK) at the external side of proteoliposomes (left) and corresponding signal intensities (right) demonstrate the inside-out orientation of the reconstituted protein. (**c**) Purified MsbA-WT and MsbA-MD preparations for reconstitution experiments were subjected to LC–MS/MS mass spectrometry. Mascot protein coverage maps for MsbA-WT and MsbA-MD are shown. The sequence stretches in red correlate to the peptides that were identified in these experiments. The MsbA-MD sequence shows a lack of peptides identified along the NBD stretch of full-length MsbA-WT. Data represent observations in three or more independent experiments with independently prepared batches of proteoliposomes. Values in histograms are expressed as mean±s.e.m. (a, one-way analysis of variance; b, unpaired student-*t* test; ***P<0.001).

To investigate the dependence of transport activity of MsbA on the electrochemical proton gradient in the absence of nucleotides and other components, MsbA-WT, MsbA-MD, MsbA-ΔK382 and the transport-inactive triple mutant MsbA-DED (D41N in transmembrane helix (TMH) 1, E149Q in TMH 3 and D252N in TMH 5) were affinity-purified and reconstituted in proteoliposomes prepared from *E. coli* phospholipids^7^,^24^. Unlike whole cells, spheroplasts and plasma membrane vesicles, these proteoliposomes are devoid of cytoplasmic constituents and alternative primary-active and secondary-active transporters, allowing studies on the transport and energetics of purified MsbA proteins in the absence of energy-transducing transport processes. The MsbA proteins incorporated equally well in proteoliposomes and were present in an inside-out orientation (Fig. 2a,b). Purified MsbA-WT and MsbA-MD samples used for the reconstitution experiments were examined by LC-MS/MS mass spectrometry. This analysis confirmed the lack of the native NBD in the MsbA-MD protein (Fig. 2c). The Mascot database was also searched against the UniProt *L. lactis subsp. lactis* database, which demonstrated insignificant levels of contaminating membrane transporters and ABC NBDs (Supplementary Data 1), below 0.01% for MsbA-WT and 0.7% for MsbA-MD when the exponentially modified protein abundance index was used as a measure for the protein abundance^25^.

**Figure 3:**
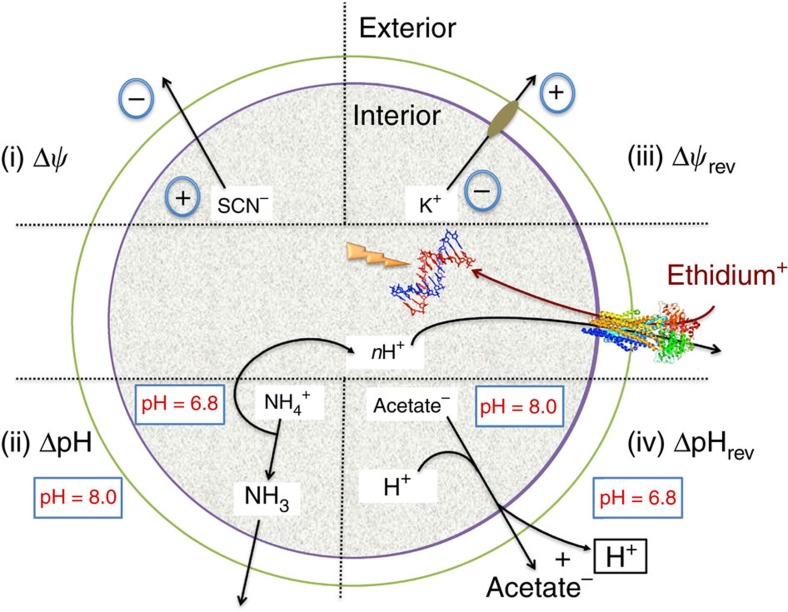
Schematic showing methods for artificial imposition of electrochemical ion gradients in proteoliposomes. The Δ*ψ* (interior positive), ΔpH (interior acidic), Δ*p* (interior positive and acidic), Δ*ψ*_REV_ (interior negative), Δ*p*_Δ*ψ*REV_ (interior negative and acidic), or ΔpH_REV_ (interior alkaline) were imposed by 100-fold dilution of proteoliposomes containing inside-out oriented MsbA-WT or MsbA-MD as described in the main text. Inclusion of DNA in the lumen allows the recording of the fluorescence emission of accumulated ethidium.

**Figure 4:**
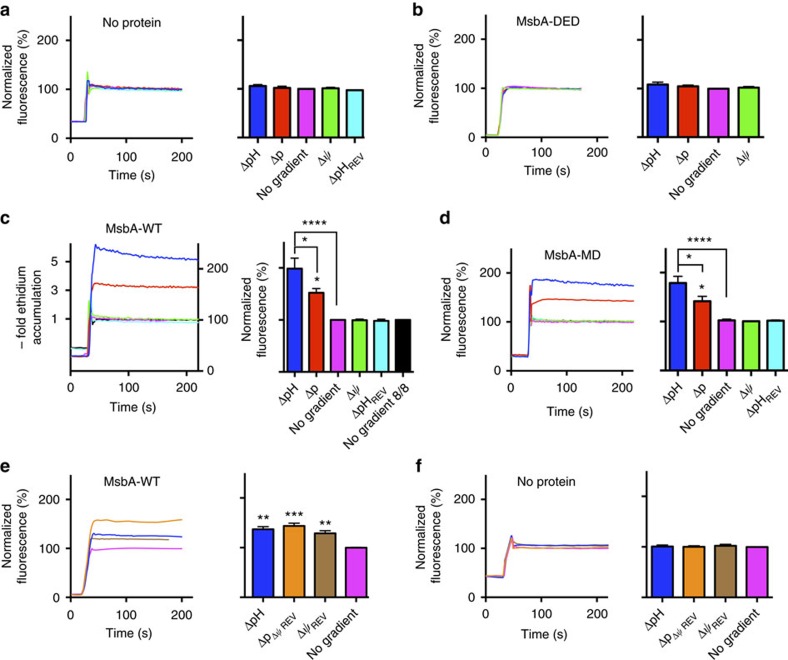
Ethidium transport in proteoliposomes. (**a**–**d**) Ethidium transport in DNA-loaded empty liposomes (**a**) or proteoliposomes containing the MsbA-DED triple mutant (**b**), MsbA-WT (**c**) or MsbA-MD (**d**) with imposed ΔpH (pH_in_ 6.8/pH_out_ 8.0), Δ*ψ* (interior positive), proton-motive force (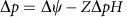 in which *Z* equals ∼58 mV at 20 °C), ΔpH_REV_ (pH_in_ 8.0/pH_out_ 6.8), or in the absence of ion gradients (pH_in_ 6.8/pH_out_ 6.8, termed *No gradient* and pH_in_ 8.0/pH_out_ 8.0, *No gradient 8/8*). *No gradient 8/8* for (a,b,d) was very close to the *No gradient* control, and is not shown for clarity of presentation. The 5-fold accumulation of ethidium by MsbA-WT is indicated in the fluorescence versus time graph in (**c**). (**e**,**f**) Effect of the imposition of a reversed Δ*ψ*_REV_ (interior negative) without or with the ΔpH (interior acidic) (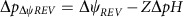) on ethidium transport in proteoliposomes containing MsbA-WT (**e**) or empty liposomes (**f**). Data represent observations in three or more independent experiments with independently prepared batches of proteoliposomes. Values in histograms show significance of fluorescence levels at steady-state, and are expressed as mean±s.e.m. (one-way analysis of variance; *P<0.05; **P<0.01; ***P<0.001; ****P<0.0001).

To study the functionality of the MsbA proteins in the proteoliposomes, a ΔpH (interior acidic) was generated by pH jump (Fig. 3). In this method, proteoliposomes prepared in buffer pH 6.8 were diluted in buffer pH 8.0, imposing a difference between the interior pH and external pH by pH jump (pH_in_ 6.8/pH_out_ 8.0). This pH difference was sustained by dissociation of NH_4_^+^ in the lumen of proteoliposomes and the outward diffusion of NH_3_. The Δ*ψ* (interior positive) was imposed by diffusion of SCN^−^ from the lumen down an outwardly directed chemical gradient ([SCN^−^]_in_/[SCN^−^]_out_=100 mM versus 1 mM). No changes in ethidium fluorescence were observed upon imposition of Δ*ψ* and/or ΔpH in liposomes lacking MsbA proteins (Fig. 4a) or containing inactive MsbA-DED (Fig. 4b). These results are consistent with the mass spectrometry data showing the absence of contaminating membrane transporters in our protein preparations (Fig. 2c and Supplementary Data 1). However, for both MsbA-WT (Fig. 4c) and MsbA-MD (Fig. 4d), ethidium transport in the proteoliposomes with the imposed ΔpH (interior acidic) was significantly higher, more than fivefold for MsbA-WT compared with the equilibration level in the no-gradient controls (pH_in_ 6.8/pH_out_ 6.8 and pH_in_ 8.0/pH_out_ 8.0). These results point to concentrative ΔpH-dependent accumulation of ethidium. In contrast, uptake of ethidium by MsbA-WT and MsbA-MD was not stimulated in the presence of a reversed ΔpH (ΔpH_REV_, interior alkaline), which was imposed by the passive diffusion of acetic acid from the lumen of the proteoliposomes (Figs 3 and 4c,d). Upon the imposition of the Δ*ψ* plus ΔpH (Δ*p*, interior positive and acidic), ethidium transport was above control but was reduced compared with the activity obtained in the presence of the ΔpH only (Fig. 4c,d). As these results suggested that the imposed Δ*ψ* (interior positive) was inhibitory for ethidium transport in proteoliposomes, the effect of reversed Δ*ψ* (Δ*ψ*_REV_, inside negative) was tested. The Δ*ψ*_REV_ was imposed in the proteoliposomes by the electrogenic downhill diffusion of K^+^ from the lumen to the external buffer by valinomycin (added at 10 nmol (mg of protein)^−1^; Fig. 3), and was found to stimulate ethidium transport in the proteoliposomes, also when combined with the ΔpH (interior acidic), yielding 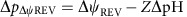 (Fig. 4e). No increase in ethidium fluorescence was observed under these conditions in liposomes lacking MsbA proteins (Fig. 4f). When taken together in the physiological context of the cell (Δ*p*, interior negative and alkaline), these findings indicate that the ΔpH (interior alkaline) supports ethidium efflux by MsbA-WT and MsbA-MD, whereas the Δ*ψ* (interior negative) inhibits this activity.

**Figure 5:**
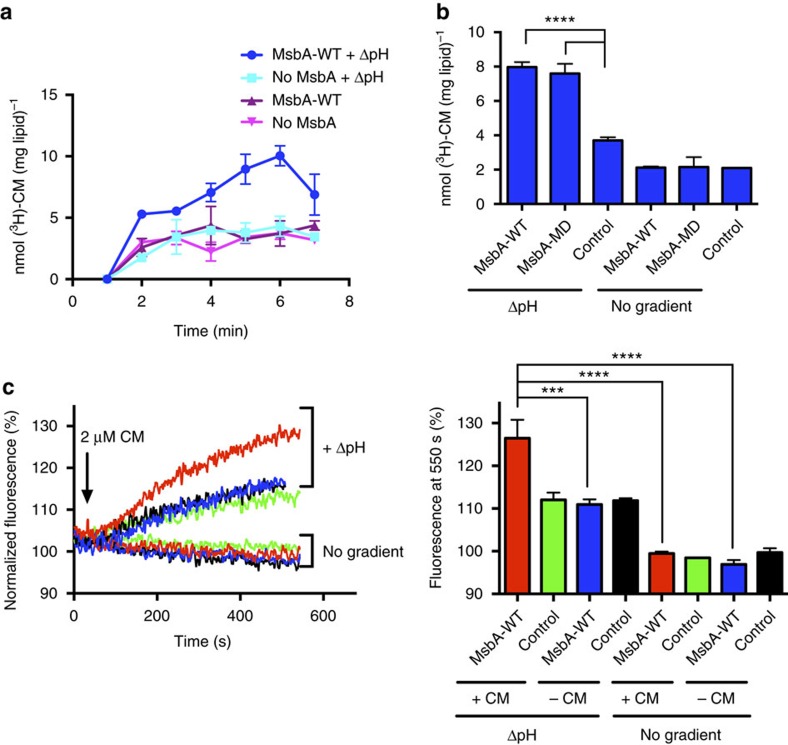
Chloramphenicol/proton antiport in proteoliposomes. (**a**,**b**) Effect of the ΔpH on the uptake of 2 μM of the neutral antibiotic [^3^H]-chloramphenicol (CM) over time (**a**) and at 5 min (**b**) in proteoliposomes containing MsbA-WT or MsbA-MD, or in liposomes without MsbA proteins. (**c**) CM uptake by in proteoliposomes is associated with H^+^ efflux through drug/proton antiport. The CM-dependent increase in fluorescence emission of the trapped pH probe BCECF in MsbA-WT-containing proteoliposomes but not in empty control liposomes indicates an increase in the lumen pH during the MsbA-WT catalysed reaction. Histogram shows BCECF fluorescence levels at 550 s. The error bars for some of the data points in (**a**) were too small to be displayed, and are hidden behind the data point symbols. Data represent observations in three or more independent experiments with independently prepared batches of proteoliposomes. Values in histograms are expressed as mean±s.e.m. (one-way analysis of variance ; ***P<0.001; ****P<0.0001).

Proton-coupled substrate transport by MsbA proteins was also observed for the neutral antibiotic chloramphenicol^20^. The 100-fold dilution of (proteo)liposomes in dilution buffer containing 2 μM [^3^H]-chloramphenicol initiated the time-dependent accumulation of chloramphenicol in proteoliposomes containing reconstituted MsbA-WT or MsbA-MD in the presence of the imposed ΔpH (interior acidic), but not in the absence of the ΔpH or in empty liposomes without MsbA proteins (Fig. 5a,b). Thus, the proton dependence of MsbA-mediated transport is observed for two different substrates, chloramphenicol and ethidium, with different charge and hydrophobicity. Using (proteo)liposomes loaded with the pH indicator 2′,7′-bis-(2-carboxyethyl)-5-(and-6)-carboxyfluorescein (BCECF), the fluorescence emission of which increases at alkaline pH, chloramphenicol uptake by MsbA-WT in the proteoliposomes was found to be associated with proton efflux down its chemical gradient (pH_in_ 6.8/pH_out_ 8.0; Fig. 5c). This result is consistent with the no-gradient control experiments in Fig. 4c in which ethidium transport by MsbA-WT in proteoliposomes was not initiated by changes in local pH in the lumen or external buffer (pH_in_ 6.8/pH_out_ 6.8 and pH_in_ 8.0/pH_out_ 8.0) but required the imposition of a transmembrane ΔpH (interior acidic).

### Proton coupling is functionally linked to ATP hydrolysis

**Figure 6:**
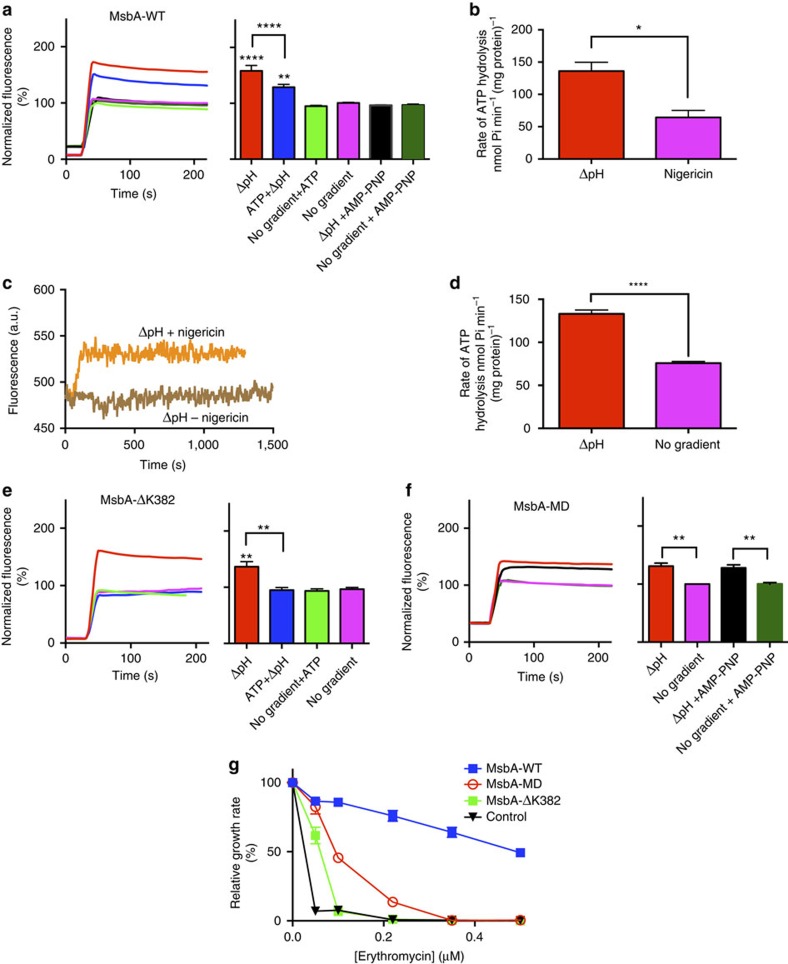
Relationship between ATP dependence and proton coupling by MsbA proteins. (**a**) Effect of the presence of 2.5 mM Mg-ATP or non-hydrolyzable nucleotide analogue AMP-PNP on imposed ΔpH (pH_in_ 6.8/pH_out_ 8.0)-dependent ethidium transport by MsbA-WT in DNA-loaded proteoliposomes. Histogram shows significance of fluorescence levels at steady-state. (**b**,**c**) MsbA-WT ATPase activity in proteoliposomes in which the ΔpH (pH_in_ 6.8/pH_out_ 8.0) was dissipated in the presence of nigericin (leading to pH_in_ 8.0/pH_out_ 8.0) (**b**). This action of nigericin was confirmed using proteoliposomes (pH_in_ 6.8/pH_out_ 8.0) loaded with the pH probe BCECF (*brown trace*), the fluorescence emission of which was enhanced by the increase in the lumen pH from 6.8 to 8.0 by the addition of the ionophore at *t*=0 s (*orange trace*) (**c**). (**d**) MsbA-WT ATPase activity in proteoliposomes in the presence of an imposed ΔpH (pH_in_ 6.8/pH_out_ 8.0) or its absence (pH_in_ 8.0/pH_out_ 8.0). Note that the pH near the NBD of MsbA (at the external side of the proteoliposomes) remains constant in the experiments displayed in (b) and (d). (**e**,**f**) Experiments as described in (**a**) in proteoliposomes containing MsbA-ΔK382 (**e**) or MsbA-MD (**f**). (**g**) Erythromycin resistance in cells expressing MsbA-WT (blue squares), MsbA-MD (red circles), MsbA-ΔK382 (green squres) compared to non-expressing control cells (black triangles). Maximum specific growth rate (*μ*_max_) was determined at each erythromycin concentration and is presented as a percentage of *μ*_max_ in the absence of erythromycin. The error bars for some of the data points in (**g**) were too small to be displayed, and are hidden behind the data point symbols. Data represent observations in 3 or more independent experiments with independently prepared batches of proteoliposomes or cells. Values in histograms are expressed as mean±s.e.m. (one-way analysis of variance except for (**b**,**d**) unpaired student-*t* test; *P<0.05; **P<0.01; ****P<0.0001).

In view of the finding that ethidium transport by MsbA is dependent on ATP hydrolysis (Fig. 1c) and components of the Δ*p* (Figs 1f and 4c,e), the relationship between these two forms of metabolic energy was further studied in proteoliposomes. For this purpose, ethidium uptake in MsbA-WT-containing proteoliposomes was measured in the absence or presence of the imposed ΔpH (inside acidic; pH_in_ 6.8/pH_out_ 8.0) in buffer containing 2.5 mM Mg-ATP or non-hydrolysable nucleotide analogue AMP-PNP. Remarkably, the ATP did not initiate ethidium accumulation in the absence of the ΔpH, nor did the nucleotide enhance ΔpH-dependent transport (Fig. 6a). However, the ΔpH-dependent accumulation of ethidium was strongly inhibited by the replacement of ATP by the non-hydrolysable analogue AMP-PNP (Fig. 6a). These data demonstrate the importance of ATP hydrolysis by MsbA-WT in experiments where ATP is co-applied with the imposed ΔpH. In agreement with this, measurements of the MsbA-ATPase activity in proteoliposomes showed that imposition of the ΔpH (pH_in_ 6.8/pH_out_ 8.0) stimulated the ATPase activity of MsbA-WT compared with controls in which the ΔpH was dissipated through the addition of nigericin (pH_in_ becomes equal to pH_out_=8.0; Fig. 6b,c) or in which the ΔpH was not imposed (pH_in_/pH_out_ set at 8.0/8.0; Fig. 6d). In these experiments, the local pH near the MsbA-NBD at the external side of the membrane remained constant. These data suggest that the conformational changes in MsbA-WT associated with ethidium transport can occur in the absence of ATP in a reaction driven by a ΔpH and Δ*ψ*_REV_. However, when ion gradients are imposed in the presence of ATP, proton coupling becomes functionally linked to ATP binding and hydrolysis, which are required to drive the dimerization and dissociation of the NBDs during the propagation of the transport cycle. Although the MsbA-ΔK382 mutant can operate in a ΔpH-dependent manner in the absence of ATP, the addition of ATP traps this mutant in an ATP-bound state and renders it transport-inactive (Fig. 6e) in an analogous manner as observed in ATP-containing cells (Fig. 1c,d). This inhibitory trapping was mimicked by the addition of the non-hydrolysable AMP-PNP to MsbA-WT (Fig. 6a). The inhibitory effect of AMP-PNP on ΔpH-dependent ethidium accumulation in the proteoliposomes was not observed for MsbA-MD lacking the NBD (Fig. 6f).

### MsbA-WT is more efficient than MsbA-MD

The observations on active drug transport by MsbA-MD raise questions about the functional importance of ATP binding and hydrolysis in full-length MsbA. The direct comparison of the transport activities of MsbA-WT and MsbA-MD in cells show that MsbA-WT catalyses ethidium efflux to lower intracellular steady-state levels than MsbA-MD (Fig. 1c,d). When cell growth was measured in the presence of the MsbA substrate erythromycin^15^, MsbA protein expression caused significant shifts in the erythromycin concentration at which the growth rate is half-maximal (IC_50_), from 0.004 μM or the non-expressing control to 0.393 μM (*P*=0.008) for MsbA-WT and 0.094 μM (*P*=0.003) for MsbA-MD; the IC_50_ for MsbA-ΔK382 (0.051 μM) was close to control (Fig. 6g). Hence, the enhanced efficiency of efflux by full-length MsbA compared with the NBD-less protein was also found in the ability of the MsbA proteins to confer cellular resistance to the antibiotic erythromycin. The ATP-dependent dimerization of the NBDs with closure of the substrate-binding cavity towards the inside surface of the membrane facilitates capture of substrate from the cellular interior and/or inner membrane leaflet, and enables efflux against a larger drug concentration gradient and/or lipid–water partition coefficient. The ATP dependence therefore enhances the directionality of the transport reaction.

## Discussion

Although MsbA is an ABC transporter that mediates substrate transport in an ATP-dependent manner, the experiments in intact cells and proteoliposomes prepared from *E. coli* phospholipids demonstrate for the first time that the ATP-binding-associated power stroke during drug transport is assisted by proton coupling via apparent drug–proton antiport. The dissipation of the ΔpH (interior alkaline) in cells by the addition of nigericin blocks MsbA-WT-mediated ethidium efflux (Fig. 1f). Conversely, the artificial imposition of the ΔpH (inside acidic) in proteoliposomes initiates (i) the accumulation of ethidium and chloramphenicol by purified, inside-out oriented MsbA-WT above the equilibration level (Figs 4c and 5a,b) and (ii) proton efflux in a chloramphenicol-dependent manner (Fig. 5c). The role of the ΔpH in MsbA-mediated transport in cells is also supported by the observations on the erythromycin efflux by MsbA-MD against the inwardly directed drug concentration gradient that impairs growth of the non-expressing control cells (Fig. 6g). Proton-coupled ethidium efflux by MsbA-WT is inhibited by the Δ*ψ* (interior negative) in cells (Fig. 1f) and equivalent Δ*ψ* (interior positive) in the proteoliposomes (Fig. 4c). Together with the observed stimulation of transport in proteoliposomes by the Δ*ψ*_REV_ (interior negative; Fig. 4e), the data point to apparent electrogenic antiport of ethidium^+^ and *n*H^+^ with *n*<1. Thus, two or more ethidium^+^ molecules are exchanged per H^+^, which is consistent with the presence in this type of ABC transporter of two cavities at the MD–MD interface that are related by twofold pseudosymmetry and that can be separated by mutation^10^,^24^,^26^,^27^. Proton-coupled transport is associated with the MD of MsbA; the observations on ΔpH dependence for MsbA-WT in intact cells and proteoliposomes could all be reproduced using MsbA-MD that lacks the NBD (Figs 1e and 4d).

Evidence was obtained that proton coupling operates in conjunction with a functional catalytic cycle at the NBDs when nucleotide is present. First, ethidium efflux in metabolically active cells containing mM concentrations of ATP^21^ was inhibited by the MsbA-ΔK382 mutation (Fig. 1c). As the NBDs are conformationally coupled to the MDs, the reduced rate of ATP hydrolysis in the mutant will cause more persistent binding of the nucleotide, which in turn will block the propagation of the catalytic cycle, and, hence, inhibit transport. Second, for MsbA-WT this transport reaction in proteoliposomes was significantly inhibited by the inclusion of the non-hydrolysable ATP analogue AMP-PNP in the external buffer (Fig. 6a). Third, ΔpH (interior acidic)-dependent ethidium accumulation in proteoliposomes by MsbA-ΔK382 was inhibited by the addition of Mg-ATP to the external buffer where the NBDs reside (Fig. 6e). Fourth, the addition of ATP or AMP-PNP had no effect on ΔpH (interior acidic)-dependent ethidium accumulation in proteoliposomes containing MsbA-MD without the NBD (Fig. 6f). Finally, the imposition of a ΔpH stimulated the MsbA-WT ATPase activity in proteoliposomes (Fig. 6b–d). The dependence of drug transport on the genotype of the expressed or reconstituted MsbA proteins demonstrates that the drug transport activity is not dependent on auxiliary proteins but on MsbA itself. This conclusion is consistent with the mass spectrometry analysis demonstrating insignificant levels of contaminating membrane transporters or NBDs in our protein preparations (Fig. 2c).

Protons can have different roles in the mechanisms of membrane transporters. A role of H^+^ in primary-active transport was previously described for the P-type Ca^2+^-ATPase (SERCA), in which protons neutralize Ca^2+^-coordinating carboxylates following substrate dissociation, essentially giving primary-active transmembrane proton-Ca^2+^ antiport^28^,^29^. H^+^ binding and movement in proton-coupled secondary-active transporters are also known to induce changes in electrostatic and hydrogen-bonding interactions between interhelix side chains that underlie the conformational transitions associated with proton–substrate symport and antiport^30^,^31^. The finding of proton-coupled transport by MsbA suggests that similar mechanistic principles are relevant for ABC exporters. Indeed, recent structural studies on the antibacterial peptide ABC exporter McjD from *E. coli* conclude that the conformational transitions required for substrate transport might not all be dependent on ATP binding and hydrolysis^32^. The MsbA data share similarities with observations on the dual mode of energy coupling by the arsenite and antimonite-translocating ArsB protein from *E. coli*^33^,^34^, which acts as a secondary-active metalloid–proton antiporter, but when associated with the ArsA ATPase subunit can utilize ATP for improved extrusion efficiency. The findings for MsbA-MD are reminiscent to those described for the MD of the ABC exporter LmrA from *L. lactis*, which catalyses apparent ethidium–proton symport^22^,^35^, illustrating that the coupled transport of substrate and protons is more widespread among ABC exporters. Our conclusions introduce proton coupling as a new parameter in the mechanism of MsbA, and point to the existence of proton-coupled conformational transitions in its transport cycle. This work is of fundamental importance for our understanding of how ABC exporters operate.

## Methods

### Bacterial strains and plasmids

The drug-hypersensitive *L. lactis* strain NZ9000 Δ*lmrA* Δ*lmrCD* strain devoid of the endogenous ABC multidrug transporters LmrA and LmrCD^36^,^37^ was used as a host for expression vector pNZ8048-derived plasmids^36^ that contain a chloramphenicol resistance marker gene, nisin-inducible *nisA* promoter and His-tagged wild-type (WT) or mutant MsbA gene, or truncated MsbA gene encoding the MD only.

### Construction of MsbA mutants

To express N terminally His-tagged MsbA-MD, the corresponding region of the *msbA* gene from *E. coli* was PCR-amplified from pNZMsbA^15^ with the forward primer 5′- GGAGGCACTCACCATGGGC -3′ and the reverse primer 5′- CGGATAAGTTCTAGATTAATTGCGGAATTCCACGTCGGC -3′ to insert a TAA stop codon after the codon for N346, equivalent to H353 in previous work on LmrA^22^, followed by an XbaI site at the 3′ end. NcoI and XbaI (Roche Applied Science, Herts, UK) were used to digest the PCR product, which was followed by ligation of the DNA fragment into the linearized vector pNZ8048 downstream of the *nisA* promoter, yielding pNZMsbA-MD. For the generation of pNZMsbA-DED (D41N E149Q D252N) the following primers were used: D41N (forward) 5′- GCCAGC**AAC**ACCTTCATGTTATCGCTCC -3′, (reversed) 5′- AAGGT**GTT**GCTGGCTGCGTTGAGGATTA -3′; E149Q (forward) 5′- TGTGCGT**CAA**GGTGCGTCGATCATCGGC -3′, (reversed) 5′- ACGCACC**TTG**ACGCACAACAGTAATCAG -3′; D252N (forward) 5′- CATCTCT**AAT**CCGATCATTCAGCTGATC -3′, (reversed) 5′- TGATCGG**ATT**AGAGATGGAAGAGGCTGA -3′. The DNA was sequenced to ensure that only the intended changes were introduced.

### Growth conditions and protein expression

*L. lactis* NZ9000 Δ*lmrA* Δ*lmrCD* was grown overnight in M17 medium (Difco) supplemented with 0.5% glucose and 5 μg ml^−1^ chloramphenicol at 30 °C to an OD_660_ of 0.5–0.6. For protein expression, cells harbouring pNZMsbA, pNZMsbA-MD, pNZMsbA-ΔK382 (ref. 16), pNZMsbA-DED or pNZ8048 (empty vector) were incubated for 1 h at 30 °C in the presence of a 1:1,000 dilution of the culture supernatant of nisin-A producing *L. lactis* strain NZ9700, corresponding to a nisin A concentration of ∼10 pg ml^−1^ (ref. 38), unless stated otherwise.

### Ethidium transport in de-energized cells

*L. lactis* NZ9000 Δ*lmrA* Δ*lmrCD* cells expressing MsbA, MsbA-ΔK382 or MsbA-MD and non-expressing control cells were grown to an OD_660_ of 0.6, and protein expression was induced for 1 h at 30 °C by 10 pg ml^−1^ nisin A. Cell pellets from 50 ml culture were harvested by centrifugation (6,500*g* for 10 min at 4 °C) and washed with ice-cold washing buffer (50 mM KPi, pH 7.0, containing 5 mM MgSO_4_). To deplete intracellular ATP levels, cells were incubated with 0.5 mM of the protonophore 2,4-dinitrophenol for 30 min at 30 °C. The protonophore was removed by centrifugation, followed by washing of cells with the washing buffer. Finally, the cells were resuspended in washing buffer to an OD_660_ of 5.0. For each measurement, cells were diluted at 1:10 into 2 ml washing buffer in a glass cuvette. Fluorescence was followed in a LS 55B Luminescence Spectrometer (PerkinElmer, MA, USA) at excitation and emission wavelengths of 500 and 580 nm with slit widths of 10 and 5 nm, respectively. Owing to differences in the uptake rates, ATP-depleted control cells and cells containing MsbA-WT or MsbA-MD were pre-loaded with 2 μM ethidium bromide for 50, 25 and 30 min, respectively, to a similar starting fluorescence level. Active efflux was subsequently initiated by the addition of 0.5% glucose, which re-energized the cells and fluorescence was followed for ∼10 min. To further determine the influence of the magnitude and composition of the Δ*p* on MsbA-mediated transport, ionophores nigericin and valinomycin were added (final concentration 1 and 0.1 μM, respectively) before activation of cells. When cells are suspended in high K^+^ containing buffer, nigericin mediates the antiport of H^+^ and K^+^ down their concentration gradients, thereby selectively dissipating *Δ*pH in an electroneutral manner. Furthermore, valinomycin mediates electrogenic uniport of K^+^, allowing the electrophoretic uptake of K^+^ in cells with dissipation of the *Δψ* (ref. 39).

### Preparation of inside-out membrane vesicles

Inside-out membrane vesicles were prepared from *L. lactis* NZ9000 Δ*lmrA* Δ*lmrCD* cells harbouring pNZ8048-based expression vectors using cell disruption equipment^15^. For this purpose, lactococcal cells were grown at 30 °C to an OD_660_ of 0.6–0.8 and incubated for 1 h in the presence of nisin A to induce protein expression. Cells were then harvested by centrifugation at 13,000*g*, 12 min, 4 °C and washed with ice-cold 100 mM KPi (pH 7.0) or 100 mM K-HEPES (pH 7.0) when the vesicles were prepared to measure ATPase activity. The cell pellet was resuspended in 20 mM KPi/K-HEPES containing Complete-Protease Inhibitor Cocktail (Roche) followed by the addition of 3 mg ml^−1^ lysozyme (from chicken egg white) and further incubation for 30 min at 30 °C. Cell lysis was achieved by passage twice through a Basic Z 0.75 kW Benchtop Cell Disruptor (Constant Systems, Northlands, UK) at 20 kpsi. The suspension was supplemented with 10 μg ml^−1^ DNase and 10 mM MgSO_4_ andincubated for 30 min at 30 °C to remove DNA. Subsequently, 15 mM K-EDTA (pH 7.0) was added to prevent the aggregation of membrane vesicles. A low spin at 13,000*g* for 40 min was performed to remove cell debris and whole cells. Membrane vesicles were harvested from the supernatant by ultra-centrifugation at 125,000*g* for 1 h at 4 °C. The membrane vesicles were resuspended in 50 mM KPi/K-HEPES (pH 7.0) containing 10% glycerol and stored as 500 μl aliquots in liquid nitrogen. The expression of MsbA proteins in membrane vesicles was assessed on Coomassie-stained SDS–PAGE, and immunoblots probed with primary mouse anti-polyhistidine tag antibody (Sigma-Aldrich, cat. no.: H1029) and secondary goat antimouse antibody (Sigma-Aldrich, cat. no.: A4416) were used at dilutions of 1:1,000 and 1:5,000, respectively (Supplementary Fig. 1).

### Purification of His-tagged MsbA proteins

His-tagged MsbA proteins were purified from membrane vesicles by Ni^2+^-nitrilotriacetic acid (NTA) affinity chromatography^7^,^24^,^38^. Membrane vesicles (diluted to 5 mg ml^−1^) were solubilized in buffer containing 50 mM KPi or K-HEPES (pH 8.0), 10% (v/v) glycerol, 0.1 M NaCl and 1% (w/v) n-dodecyl-β-D-maltoside (DDM) (Melford Laboratories Ltd., UK) for 4  h by gently mixing on a rotating wheel at 4 °C. Insoluble particles were removed by centrifuging the mixture at 125,000*g*, 4 °C for 40 min. The solubilized protein was mixed with Ni^2+^-NTA resin at a ratio of 10 mg His-tagged protein per ml of resin. The resin was pre-equilibrated by washing thrice with five resin volumes of Milli Q water and twice with five resin volumes of wash Buffer A (50 mM KPi or K-HEPES (pH 8.0), 0.1 M NaCl, 10% (v/v) glycerol, 0.05% (w/v) DDM and 20 mM imidazole). The suspension was left on a rotating wheel for overnight binding at 4 °C, after which the resin was collected by centrifugation and transferred to a 2 ml volume Biospin disposable chromatography column (Bio-Rad). After subsequent washing with 20 volumes of wash Buffer A and 30 volumes of wash Buffer B (50 mM KPi or K-HEPES (pH 7.0), 0.1 M NaCl, 10% (v/v) glycerol, 0.05% (w/v) DDM and 20 mM imidazole added from 1 M imidazole stock (pH 8.0)), His-tagged protein was eluted with 3–4 volumes of Elution Buffer (50 mM KPi or K-HEPES (pH 7.0), 0.1 M NaCl, 5% (v/v) glycerol, 0.05% (w/v) DDM and 150 mM imidazole added from 1 M imidazole stock (pH 8.0)). The eluted purified protein was kept on ice and was immediately used for experiments. Purity of the protein was monitored on a 10% SDS–PAGE with Coomassie Brilliant Blue staining.

### LC–MS/MS mass spectrometry analysis

Purified MsbA proteins were reduced (DTT) and alkylated (iodoacetamide) and subjected to enzymatic digestion with trypsin overnight at 37 °C. Aliquots were then pipetted into a sample vial and loaded onto an autosampler for automated LC–MS/MS analysis^40^. All LC–MS/MS experiments were performed using a nanoAcquity UPLC (Waters Corp., Milford, MA) system and an LTQ Orbitrap Velos hybrid ion trap mass spectrometer (Thermo Scientific, Waltham, MA). Separation of peptides was performed by reverse-phase chromatography using a Waters reverse-phase nanocolumn (BEH C18, 75 μm inner diameter × 250 mM, 1.7 μm particle size) at flow rate of 300 nl min^−1^. Peptides were initially loaded onto a pre-column (Waters UPLC Trap Symmetry C18, 180 μm inner diameter × 20 mm, 5 μm particle size) from the nanoAcquity sample manager with 0.1% formic acid for 5 min at a flow rate of 5 μl min^−1^. After this period, the column valve was switched to allow the elution of peptides from the pre-column onto the analytical column. Solvent A was water+0.1% formic acid and solvent B was acetonitrile+0.1% formic acid. The linear gradient employed was 5–40% B in 40 min (total LC–MS/MS run time was 60 min). The LC eluant was sprayed into the mass spectrometer by means of a New Objective nanospray source. All *m/z* values of eluting ions were measured in the Orbitrap Velos mass analyser, set at a resolution of 30,000. Data dependent scans (Top 20) were employed to automatically isolate and generate fragment ions by collision-induced dissociation in the linear ion trap, resulting in the generation of MS/MS spectra. Ions with charge states of 2+ and above were selected for fragmentation. Post run, the data were processed using Protein Discoverer (version 1.4., ThermoFisher). Briefly, all MS/MS data were converted to mgf files and these files were then submitted to the Mascot search algorithm (Matrix Science, London, UK) and searched against the Uniprot *L. lactis subsp. lactis* strain IL1403 database (taxon identifier 1360) using a fixed modification of carbamidomethyl (C), variable modifications of oxidation (M) and deamidation (NQ). The peptide mass tolerance was set to 25 ppm, the fragment ion mass tolerance to 0.8 Da and the maximum number of missed cleavages to 2.

### Reconstitution of purified MsbA proteins

Purified protein (MsbA-WT, MsbA-ΔK382, MsbA-DED or MsbA-MD) was reconstituted in proteoliposomes prepared from acetone–ether-washed *E. coli* phospholipids^7^,^38^,^41^ diluted to 4 mg ml^−1^ in chloroform, which were mixed in a ratio of 3:1 (w/w) with egg-yolk phosphatidylcholine (Avanti Polar Lipids Inc.). Solvent was evaporated using N_2_ gas after which the lipid mixture was rehydrated using Buffer 1 (10 mM K-HEPES (pH 6.8), 10 mM Tris-Cl, 100 mM K_2_SO_4_ and 100 mM NH_4_SCN) or Buffer 2 (10 mM Tris-Cl (pH 8.0), 10 mM K-HEPES and 100 mM KSCN; see under ‘Substrate transport in proteoliposomes'), and 1 mg ml^−1^ of sonicated calf thymus DNA (Trevigen) for ethidium transport measurements. After resuspension, lipids were extruded 11 times through a 400-nm polycarbonate filter to form unilamellar liposomes of homogenous size and destabilized by the step-wise addition of Triton X-100 which was followed at OD_540_ (ref. 38). For reconstitution, purified protein was mixed with the detergent-destabilized liposomes in a 1/50 ratio (w/w) and incubated at room temperature (RT) for 30 min. Detergent was then removed using polystyrene bio-beads (Bio-Bead SM-2, Bio-Rad). For this purpose, Bio-Beads were pre-washed three times with methanol, once with ethanol and five times with water before use. Successive extractions of detergent were achieved by incubating proteoliposomes, first with 80 mg ml^−1^ Bio-Beads for 2 h at RT, then with 8 mg ml^−1^ Bio-Beads for 2 h at 4 °C and finally with 8 mg ml^−1^ Bio-Beads for 18 h at 4 °C. Proteoliposomes were harvested by centrifugation (130,000*g* for 30 min, 4 °C), resuspended in 3 ml Buffer 1 or 2, in which the liposomes were prepared, and incubated at 30 °C for 20 min with 10 mM MgSO_4_ and 10 μg ml^−1^ DNase to remove any DNA contamination from the lipid bilayer. Finally, liposomes were harvested by centrifugation (130,000*g* for 30 min, 4 °C), resuspended in 150–200 μl Buffer 1 or 2 that was used for their preparation and used immediately for transport studies. Samples were maintained on ice.

### Substrate transport in proteoliposomes

Ethidium transport measurements with reconstituted proteoliposomes containing MsbA proteins were initiated by the 100-fold dilution of DNA-loaded proteoliposomes in 2 ml of external buffer in a 3-ml fluorescence cuvette (to a final concentration of 20 μg membrane protein per ml) to impose different electrochemical ion gradients. For this purpose, proteoliposomes in Buffer 1 (see under ‘Reconstitution of purified MsbA proteins') were diluted 100-fold in Buffer i (10 mM K-HEPES (pH 8.0), 10 mM Tris-Cl and 100 mM K_2_SO_4_) to impose the Δ*p* (interior positive and acidic), Buffer ii (10 mM K-HEPES (pH 8.0), 10 mM Tris-Cl and 100 mM KSCN) to impose the ΔpH (interior acidic), or Buffer iii (10 mM K-HEPES (pH 6.8), 10 mM Tris-Cl, 50 mM (NH_4_)_2_SO_4_ and 100 mM K_2_SO_4_) to impose the Δ*ψ* (interior positive). In experiments with the Δ*ψ*_REV_, proteoliposomes in Buffer 2 (see under ‘Reconstitution of purified MsbA proteins') were diluted 100-fold into Buffer iv (10 mM NMG-HEPES (pH 6.8), 10 mM Tris-Cl and 50 mM (NH_4_)_2_SO_4_) in the presence of 10 nmol per mg protein of valinomycin to impose the Δ*ψ*_REV_ (interior negative), Buffer v (10 mM K-HEPES (pH 8.0), 10 mM Tris-Cl and 50 mM K_2_SO_4_) to impose the ΔpH (interior acidic), or Buffer vi (10 mM NMG-HEPES (pH 8.0) and 10 mM Tris-Cl) in the presence 10 nmol per mg protein valinomycin to impose the Δ*p*_Δ*ψ*REV_ (interior negative and acidic). After 30 s of recording, ethidium bromide (2 μM) was added and fluorescence was measured as a function of time in an LS 55B luminescence spectrometer (Perkin-Elmer Life Sciences) with excitation and emission wavelengths of 500 and 580 nm with slit widths of 10 and 5 nm, respectively. In control experiments, proteoliposomes were diluted 100-fold in the buffer in which they were prepared (pH_in_ 6.8/pH_out_ 6.8 and pH_in_ 8.0/pH_out_ 8.0) to measure ethidium transport in the absence of ion gradients. In addition, empty liposomes were prepared with nickel NTA elution buffer instead of purified protein, and these were diluted in the same buffers as the proteoliposomes.

For measurements of ΔpH (interior acidic)-dependent chloramphenicol transport, proteoliposomes were generated as described for ethidium transport, but without DNA in the internal lumen, and diluted 100-fold (to 30 μg phospholipid per ml) in 500 μl dilution buffer in glass tubes containing 2 μM [^3^H]-chloramphenicol (3.33 TBq mol^−1^) (Sigma). At given time intervals, samples were withdrawn, diluted with 2 ml of ice-cold 0.1 M lithium chloride, filtered immediately through cellulose nitrate filters (0.45 μm pore size) and washed once with 2 ml of the lithium chloride solution. Radioactivity retained on the filters was measured by liquid scintillation counting. Transport data were corrected for binding of chloramphenicol to the nitrocellulose filters. To provide evidence for proton-coupled chloramphenicol transport by MsbA-WT, (proteo)liposomes were prepared in Buffer 1 (interior acidic; pH 6.8) containing 100 μM of the pH indicator BCECF (Molecular Probes). These (proteo)liposomes were diluted 100-fold in Buffer ii (pH 8.0) to impose a H^+^ gradient, or Buffer 1 for control measurements in the absence of an ion gradient. BCECF fluorescence was measured with wavelengths for excitation at 502 nm and emission at 525 nm, and with slit widths of 10 and 15 nm, respectively. Experiments were performed in triplicate using independent batches of proteoliposomes.

### ATPase activity in proteoliposomes

The ATPase activity of MsbA was monitored in reconstituted proteoliposomes in the absence or presence of the ΔpH, using the Malachite Green assay to measure the liberation of Pi over time^37^,^42^. Proteoliposomes prepared in Buffer 1 were diluted 20-fold in Buffer ii as described under ‘Substrate transport in proteoliposomes' (Fig. 6b). To dissipate the ΔpH, 1 μM nigericin was added immediately after dilution of proteoliposomes, and the mixture was kept on ice for 5 min before the measurements of ATPase activity. The ATPase reaction was started by the addition of 2.5 mM Mg-ATP (high grade ATP from Sigma), after which Pi release was measured at 1 and 2 min. Following incubation at 30 °C, the reactions were stopped by mixing 30-μl aliquots with activated malachite green-ammonium molybdate for 5 min in a 96-well plate. Samples were subsequently incubated for 25 min with 34% citric acid after which the OD_600_ was determined. Pi release between *t*=1 min and *t=*2 min was calculated. To confirm that nigericin was able to dissipate the ΔpH, 100 μM BCECF was added to the preparation Buffer 1 to include the probe in the lumen of the proteoliposomes (Fig. 6c). BCECF fluorescence emission was measured in a LS 55B luminescence spectrometer with excitation and emission wavelengths of 535 and 590 nm with slit widths of 10 and 5 nm, respectively. For the experiments in Fig. 6d, proteoliposomes prepared in buffer (pH 6.8 or 8.0) containing 10 mM Tris-Cl and 10 mM K-HEPES were diluted 20-fold in buffer (pH 8.0) containing 10 mM Tris-Cl and 10 mM K-HEPES.

### Orientation of MsbA in the membrane

Right-side-out membrane vesicles were prepared by osmotic lysis of cells^43^. MsbA-WT expressing lactococcal cells from 1 l culture were collected by centrifugation at 13,000*g* for 15 min at 4 °C. The pellet was washed once in 100 mM KPi (pH 7.0). Cells were resuspended in 5 ml of the same buffer containing half a tablet of complete protease inhibitor cocktail (Roche Applied Science), 10 mM MgSO_4_, 40 mg ml^−1^ lysozyme, and were incubated for 30 min at 30 °C under mild shaking. The protoplast suspension was mixed with 4.8 ml of a 0.75 M K_2_SO_4_, 10μg ml^−1^ DNase and RNase while stirring, and incubated for 2 min at 30 °C. The homogenized, concentrated protoplast suspension was poured directly into 36 ml 100 mM KPi (pH 7.0). The lysate was incubated for 20 min at 30 °C with vigorous swirling. K-EDTA, pH 7.0, was then added to 20 mM final concentration, and the incubation was continued for 10 min at 30 °C. Shortly after the addition of EDTA, the turbidity of the suspension decreased and the viscosity increased. Finally, MgSO_4_ was added to a final concentration of 15 mM and the incubation was continued for another 15 min at 30 °C; during this period the viscosity decreased. The lysates were centrifuged at 48,200*g* for 30 min at 4 °C. The pellet was resuspended in 48 ml 50 mM KPi buffer, pH 7.0, containing 10 mM MgSO_4_. The sample was centrifuged at 750*g* for 60 min at 4 °C and the yellowish, milky, supernatant fluid was carefully decanted and centrifuged at 48,200*g* for 30 min at 4 °C. The high speed pellet obtained as described above was resuspended by homogenization in 1 ml 50 mM KPi buffer (pH 7.0) containing 10% glycerol and frozen in small aliquots of 100 μl and stored in liquid nitrogen.

The orientation of MsbA proteins in right-side-out membrane vesicles, inside-out membrane vesicles or proteoliposomes was assessed by determining the accessibility of the N-terminal His-tag to digestion by protease K in the external buffer^41^. Membrane proteins were diluted in 50 mM K-HEPES (pH 7.0) supplemented with 1 mM CaCl_2_. The digestion was initiated by addition of proteinase K at an enzyme-membrane protein ratio of 1:25 (w/w). The samples were subsequently incubated at 0 °C for 10 min. The reaction was terminated by the addition of 10 mM phenylmethanesulphonyl fluoride (from stock in ethanol), after which 3 × SDS–PAGE sample-loading buffer and 1 mM DTT were added. The samples were incubated at RT for 10 min and analysed on immunoblot as described under ‘Preparation of inside-out membrane vesicles'.

### Cytotoxicity assays

*L. lactis* expressing MsbA-WT, MsbA-MD or MsbA-ΔK382, and non-expressing control cells were grown as described under ‘Growth conditions and protein expression' at 30 °C in 96-well plates in the presence of a range of erythromycin concentrations. Nisin A was added at a concentration of 5 pg ml^−1^ to induce protein expression, and growth was monitored by measuring OD_660_ in a Versamax plate reader (Molecular Devices Wokingham, UK) at 30 °C. The maximum specific growth rate (*μ*_m_) was determined from the change in OD_660_ over time, by fitting the data to 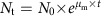 in which *N*_t_ and *N*_0_ are the cell densities at times *t* and 0 h, respectively. The *μ*_m_ of the cells grown in the absence of drug was set at 100% to calculate relative growth rates (Fig. 6g).

### Statistical analyses

Significance of data obtained with whole cells and proteoliposomes was tested by one-way analysis of variance. Differences in proteinase-K and ATPase results were assessed using the unpaired student-*t* test. Asterisks directly above bars in the histograms refer to comparisons with control; asterisks above lines refer to specific comparisons: *P<0.05; **P<0.01; ***P<0.001; ****P<0.0001.

### Data availability

Data that support the findings of this study have been deposited in the University of Cambridge data repository with the accession code 1810/255838 (https://www.repository.cam.ac.uk/handle/1810/255838) or available from the corresponding author upon reasonable request.

## Additional information

**How to cite this article:** Singh, H. *et al*. ATP-dependent substrate transport by the ABC transporter MsbA is proton-coupled. *Nat. Commun.* 7:12387 doi: 10.1038/ncomms12387 (2016).

## Supplementary Material

Supplementary Information Supplementary Figure 1

Supplementary Data 1 Summary of Mascot search results for LC-MS/MS data obtained with samples of purified MsbA-WT and MsbA-MD. These protein samples were used for reconstitution in proteoliposomes.
