# Supplementary material for: ATP-dependent substrate transport by the ABC transporter MsbA is proton-coupled
Source: Nat Commun. 2016 Aug 8;7:12387. doi: 10.1038/ncomms12387 (PMC4979069; doi:10.1038/ncomms12387)
Supplement: Supplementary Information — Supplementary Figure 1 [file ncomms12387-s1.pdf]

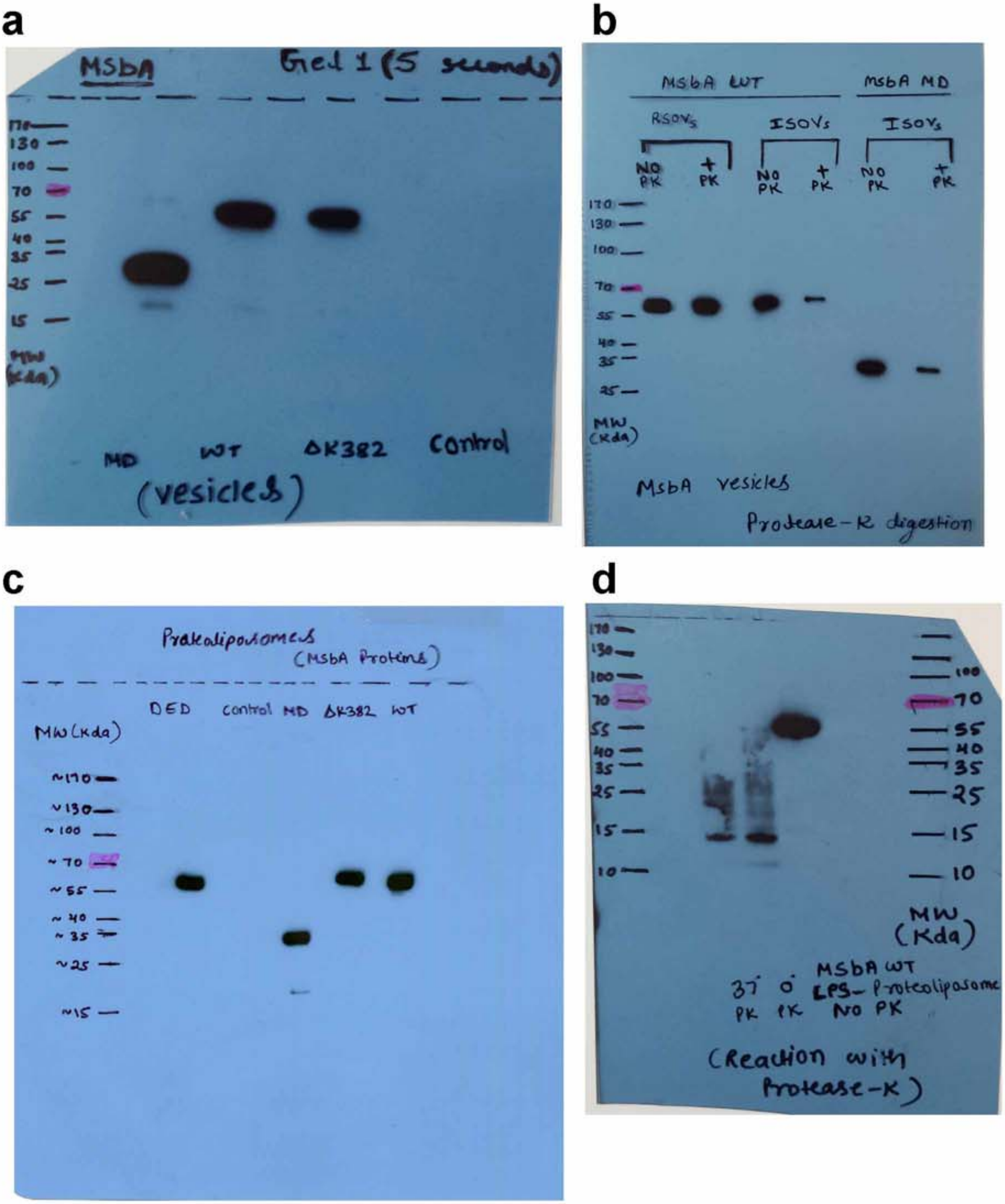

Supplementary Figure 1

(**a**) Complete immunoblot of lactococcal plasma membrane vesicles containing MsbA-MD, MsbA-WT, MsbA-ΔK382, or without MsbA protein (control), utilized in Fig. 1a. (**b**) Complete immunoblot of right side-out membrane vesicles (RSOVs) and inside-out membrane vesicles (ISOVs) containing MsbA-WT or MsbA-MD before and after exposure to protein kinase K (± PK) in the external buffer, utilized in Fig. 1b. (**c**) Complete immunoblot of proteoliposomes containing purified MsbA-DED, MsbA-MD, MsbA-ΔK382 or MsbA-WT, or without MsbA proteins (control), utilized in Fig. 2a. (**d**) Complete immunoblot of proteoliposomes containing purified MsbA-WT after exposure to PK in the external buffer at 37 ºC or 0 ºC, and before exposure to PK at 0 ºC, utilized in Fig. 2b. The migration of molecular mass markers (kDa) is indicated.
